# Mastitis impact on high-yielding dairy farm’s reproduction and net present value

**DOI:** 10.3389/fvets.2023.1345782

**Published:** 2024-01-08

**Authors:** Alina Borş, Silviu-Ionuț Borş, Viorel-Cezar Floriștean

**Affiliations:** ^1^Department of Public Health, Faculty of Veterinary Medicine, “Ion Ionescu de la Brad” Iasi University of Life Sciences, Iaşi, Romania; ^2^Research and Development Station for Cattle Breeding Dancu, Iaşi, Romania

**Keywords:** dairy cows, mastitis, reproduction, net present values, economics

## Abstract

Poor udder health can have a negative impact on milk production and reproductive performance, which reduces the net present value (NPV) of dairy farms. The aim of this study is therefore to investigate the relationship between clinical mastitis and NPV and the financial impact of impaired reproductive function. For this purpose, 473 dairy cows were included in our study, 146 cows with clinical mastitis (CM group) and 327 clinically healthy cows (CH group) from a high-yielding dairy farm in Romania, milking approximately 780 dairy cows with an average milk production of 46 kg milk/day. We found that, in contrast to CH cows, CM cows had a significantly lower conception rate at first service (58.2% vs. 41.7%, *p* < 0.05), third service (45.3% vs. 30.2%, *p* < 0.05), and total services (49.2% vs. 36.4%, *p* < 0.05). However, this positive effect was not observed for the average days open, which were significantly lower in CM cows than in CH cows (112 ± 4.3 days vs. 142 ± 3.1 days, *p* < 0.05). The fact that the non-pregnant CH cows had higher somatic cell counts (>400,000 SCC/mL) in their milk around artificial insemination (AI) and 1 month earlier than the pregnant cows (<250,000 SCC/ml) supports the idea that poor uterine health affects the reproductive activity of high-yielding cows. However, by using the UW-DairyRepro$ decision support tool, we found that despite the impairment of reproductive function in dairy cows, the largest negative impacts on NPV are still the cost of milk loss (US$14,439.4/farm/year) and treatment costs (US$4,380/farm/year). We considered the costs associated with poor reproductive function in the CM group (US$3,577/farm/year) as an additional cost of mastitis. Finally, it appears that the impact of mastitis on reproduction is associated with a lower chance of conception than it is with a daily risk of services.

## 1 Introduction

Poor reproductive performance in dairy cows is influenced by a variety of factors (1–3) such as transition cow management, metabolic and udder health, lameness, estrus detection, semen handling, and the use of synchronization protocols (4, 5). Although conception rates are significantly lower at first service, which is determined at the time of pregnancy diagnosis (2, 5, 6), fertilization rates approach 100% in heifers (6) and lower at approximately 77% in lactating dairy cows (7). Between 3.2% (8) and 42.7% (9) of pregnancies can be lost, and several factors, including heat stress, milk yield, clinical mastitis, and progesterone concentrations, have been associated with this (1, 10, 11).

Mastitis is a fairly common disease that causes high treatment costs, production losses, and milk withdrawal expenses in dairy herds worldwide (12). The timing of mastitis during insemination appears to be a factor influencing cow reproduction. For example, according to Santos et al. (6), conception rates to first service were 29% for cows never diagnosed with clinical mastitis, 22% for animals diagnosed before artificial insemination (AI), 10% for cows identified after AI, and 38% for cows diagnosed after pregnancy confirmation. Ruegg and Erskine (13) showed that the increase in somatic cell count (SCC) in milk is a reliable sign of intramammary infection. Previous studies have estimated the effect of SCC on various indicators of reproductive performance, such as longer days to first service (14–16), lower risk of conception (17–20), and higher risk of pregnancy loss (1, 15). These studies (17–20) have also reported that the most unfavorable outcome was the occurrence of mastitis events after insemination and an increase in the severity of cases. These field data are consistent with previous experimental studies (21, 22) showing how high incidences of SCC can alter the hormonal profile and lead to infertility. Hudson et al. (19) reported that mastitis episodes can last up to 60 days prior to artificial insemination, supporting the theory that pre-ovulatory oocytes can be damaged by the inflammatory response of the udder (23, 24). Furthermore, in dairy cows with reduced morula quality, conceptus elongation, and embryo survival, the effects of mastitis during early lactation have been shown to disrupt early embryo development at the pre-implantation stage (25).

An intramammary infection normally leads to an influx of inflammatory cells as a protective reaction. Cytokines play a variety of roles in inflammation. Tumor necrosis factor (TNF-), a cytokine produced in mastitis and released into the bloodstream, gets to the oviduct (26) and causes the production of prostaglandin F2α (27, 28). The smooth muscles of the oviduct contract as a result of prostaglandin F2α, which can lead to embryonic mortality in pregnant animals (29). Prostaglandin F2α not only causes the corpus luteum to regress and the progesterone level in the blood to fall but also restricts pregnancy (29). On the other hand, cytokines have been shown to have a detrimental effect on hypothalamic–pituitary function in the postpartum period, leading to abnormal gonadotropin and GnRH production (30). Due to the lack of follicular development and ovulation, reproductive problems such as anovulation after estrus, failed fertilization, and subsequent embryonic death occur (31). Pro-inflammatory cytokines are crucial in the maturation process of the ovarian follicle in addition to the process of embryo implantation in humans (32). Under physiological and pathophysiological conditions, cytokines seem to exert their pleiotropic activities in the reproductive system (33).

As far as we know, there is no international information on how mastitis affects reproduction economically. Thus, the objective of this study is to determine the net present value of mastitis in terms of milk loss, treatment costs, and impact on reproduction.

## 2 Materials and methods

### 2.1 Study design

The north-eastern Romanian Holstein-Friesian dairy herd served as the subject of this study. The average number of lactating cows in the herd during the study period was approximately 780 and the average annual milk yield per cow was 14,030 kg/305 days. Depending on their body condition score and whether or not they were pregnant with twins, dry cows were kept in a separate group and moved to a “parturition group” (close-up group) 21 days before parturition.

The cows were housed in free-stall barns with concrete floors and straw bed and fed a Total Mixed Ration twice a day with *ad libitum* water access according to the level of milk production and cow size. To keep the animals healthy, standard management practices, including a cooling system coupled with a weather station for hot months, were followed. During the study period, approximately 780 cows were milked on the farm three times a day at 04.00, 12.00, and 19.00, corresponding to a daily average of 46 kg milk/cow/day.

The investigated dairy farm performed somatic cell screening once a month to identify and determine the cows with high somatic cell content that were last milked in the milking program. The investigators examined the eligible cows and excluded those with very severe lesions at the teat ends or severe mastitis [pyrexia (>40.0°C), gross signs of dehydration or recumbency]. The affected gland was subjected to a California mastitis test (CMT), which gave results of 0, trace, 1, 2, or 3. Milk was collected from each cow milked according to standard Mastitis Council protocols (34). Before the milk samples were collected, the udder and teat opening of the dairy cow were thoroughly washed, cleaned, and dried. In addition, dirt and other contaminants were wiped off the teat and udder with a dry towel. To prevent recontamination, the teats were carefully cleaned with cotton before being immersed in 70% alcohol. First, the other side of the teats of the udder was cleaned with alcohol, and samples were taken then the near side. The milk was categorized as having no clots (0), flecks (1), or clots (2). After discarding a few milliliters of milk, the collection container was held almost horizontally to collect approximately 10 mL of milk. All procedures were carried out according to the guidelines of the National Mastitis Council. Finally, all milk samples were labeled and transported to the veterinary microbiology laboratory in an ice container (34). The samples were transported on ice between the investigators’ facilities and the farm for a maximum of 8 h. They were then stored at −20°C until a courier delivered them to the two accredited laboratories which used the matrix-assisted laser desorption/ionization time-of-flight mass spectrometry (MALDI-TOF MS) to the diagnosis of microbial infections.

During the period of the study, all dairy cows suffering from clinical mastitis were given antibiotics and non-steroidal anti-inflammatory drugs. The veterinarians on the farm were able to treat severe cases; however, if medication failed to heal the damaged gland, it was treated therapeutically. Furthermore, if necessary, other conditions were treated with additional antibiotics or medications. The farm kept records of all treatments given to the cows throughout their lactation period. In addition, electronic records of each breeding date were kept for cows enrolled in the study.

Estrous cows were identified using the AfiMilk (AfiMilk, Kibbutz Afikim, Israel) estrus daily report, and each one was examined by an experienced veterinarian. Attempts to mount other cows, chasing herdmates, restlessness, chin leaning, sniffing the vagina of herdmates and roaring, and relaxation and mucus discharge from the vulva were the signs of estrus. The manifestation of a standing estrus was considered a sign of a true estrus. According to Ciornei and Roșca (35), artificial insemination was performed by transrectal localization of the cervix and the use of the Cassou insemination gun to pass through the transcervical passage, the insemination procedure followed the traditional Anglo-Saxon method. The sperm was deposited in the ipsilateral uterine horn to the ovary with the largest follicle, paying particular attention to this. A plastic protective film was also used.

All pregnancy and mastitis diagnostic procedures were performed by a single, experienced veterinarian. Ultrasonography of the uterus and ovaries was performed using a 5–7.5 MHz rectal convex probe (BCF EasyScan, BCF Ultrasound Australasia, Mitcham, Victoria, Australia) to evaluate ovarian structure and diagnose pregnancy 30 days after artificial insemination (AI) based on visualization of a fluid-filled uterine horn and the presence of an embryo associated with a corpus luteum. Confirmation of pregnancy was performed 90 and 221 days after AI. The study was conducted on 473 dairy cows divided into two groups: clinically healthy cows (CH group, *n* = 327) and clinical mastitis cows (CM group, *n* = 146) suffering from this disease after parturition. During the study, the cows were allocated to the pens according to day in milk (DIM) and parity. Calving data, breeding data, and DIM were taken from the AfiMilk management software (AfiMilk, Kibbutz Afikim, Israel).

### 2.2 Economic analysis

A total of 146 CM dairy cows and 327 CH dairy cows from a commercial dairy herd (n = 780) with a production of 14.030 kg milk/cow/305 days were simulated using the UW-DairyRepro$ decision support tool (36) with the modifications described by Giordano et al. (37) to evaluate the economic impact of clinical mastitis on dairy cows reproduction. The reproductive program simulated for the CM group was similar to that of the CH group, with the difference that the reproductive parameters obtained were adjusted. The following herd, economic, and reproductive parameters were taken into account: average body weight (1800 lb), involuntary culling (28%), mortality rate (4%), stillbirths (4.9%), milk price (US$20/cwt), cost feed lactation (US$0.08/lb. DM), fixed cost of dry period (US$0.06/lb. DM), value of female calves (US$200), value of male calves (US$100), replacement value of heifers (US$1800), residual value (US$0.526/lb), the adjusted voluntary waiting period (105 for the CH group and 121 for the CM group), the length of the estrus cycle (30 days for the CH group and 33 days for the CM group), the maximum milk day for breeding (300 days), the minimum milk yield for non-breeding (30 kg/day), pregnancy loss (5%), day of first pregnancy check (30 days after AI), day in gestation second pregnancy check (90 days after AI), and conception rate at first service (58.1% for the CH group and 41.7% for the CM group). The costs of reproduction programs are insemination costs (semen US$20/cow and labor US$5/cow) and ultrasound pregnancy monitoring (US$100/h). The costs of a timed AI (TAI) protocol were not included for either group as the first AI postpartum was a heat breeding. The model estimated the differences in net present value (NPV, US$/cow/year) for the reproductive programs consisting of improved conception rates at first service in the CH group compared to the CM group, to which mastitis costs (diagnosis, treatments, and the destruction of mastitis milk) were added. The estimation of NPV included the average milk production of the entire herd.

### 2.3 Statistical analysis

Using a binary logistic regression (logistic procedure from PASW Statistics for Windows Version 21, SPSS Inc., Chicago, IL, United States) and considering lactation, days in milk, milk yield, type of estrus, repeat breeder, sire and technician, the effects of CM on conception rates at first service, number of AI per conception, interestrus interval, the number of services per calving, days open and pregnancy loss (dependent variables) were investigated. Estimates and Wald 95% limits were used to calculate odds ratios and 95% confidence intervals. Explanatory variables and interactions were assessed using backward elimination to ensure that only factors significantly affecting pregnancy remained in the model (38). Statistical significance was set at a *p*-value < 0.05. Values are given as mean ± standard error of the mean.

## 3 Results

A total of 149 cows were diagnosed with clinical mastitis, with 200 quarters affected from approximately 780 dairy cows.

*Pseudomonas aeruginosa, Enterobacter cloacae, E coli*, *coagulase-negative staphylococci*, *Streptococcus lutetiensis*, and *Streptococcus uberis* were the pathogens isolated from mastitis milk. Three dairy cows were eliminated from the trial despite receiving antibiotic therapy because of septicemia, a consequence of mastitis.

Table 1 shows that some reproductive indices of the CM group were significantly (*p* < 0.05) more influenced than those of the CH group. There were differences between the CM and CH groups in terms of average days to first service (*p* < 0.05), average days open (*p* < 0.05), and first service conception rate, all of which were higher in the healthy cows. The first and third conception rates and the total conception rate were also lower (*p* < 0.05) in the CM group compared to the CH group. However, no difference (*p* > 0.05) was observed for the inter-estrus intervals although the proportion of two and more than two TAI services was higher (*p* < 0.05) in the CH group compared to the CM group (Table 2).

**Table 1:** Reproductive parameters of the CM group vs. CH group.

Reproductive parameters	CM group	CH group
	(*n* = 146)	(*n* = 327)
Average days to first service	121 ± 5.1^a^	105 ± 4^b^
Average days open	112 ± 4.3^b^	142 ± 3.1^a^
Calving interval	399 ± 6.2	392 ± 4.1
The number of AI per conception	1.5 ± 0.7	1.4 ± 0.1
First insemination conception rate (%)	41.7^b^	58.1^a^
Second insemination conception rate (%)	31.2	39.1
Third insemination conception rate (%)	30.2^b^	45.3^a^
Total insemination conception rate (%)	36.4^b^	49.2^a^
Open more than 150 days (%)	17.6	14.1

Different superscripts (^a,b^) in the same row indicate significant difference (*p* < 0.05).

**Table 2:** Interestrus interval and proportion of TAI services of the CM group vs. CH group.

Parameters	CM group	CH group
	(*n* = 146)	(*n* = 327)
AI1–AI2 (M ± SEM, days)	33.5 ± 1.7	30.3 ± 0.8
AI2–AI3 (M ± SEM, days)	31.1 ± 2.9	31.9 ± 1
AI3-AI4 (M ± SEM, days)	29 ± 2.6	31.4 ± 1.3
AI4-AI5 (M ± SEM, days)	34.4 ± 6.4	30.6 ± 2.7
Proportion of one TAI service (%)	20.5	27.16
Proportion of two TAI service (%)	6.2^b^	11.04^a^
Proportion of more than two TAI service (%)	2.1^b^	3.6^a^

Different superscripts (^a,b^) in the same row indicate significant difference (*p* < 0.05).

With regard to the timing of mastitis and its effect on pregnancy at the first service, we observed a higher conception rate (*p* < 0.05) in cows diagnosed with mastitis in the 85–100 day postpartum interval compared to cows diagnosed with mastitis in the 22–84 day interval and after 100 days postpartum. On the other hand, the number of services per calving (NSC) did not differ (*p* > 0.05) depending on the time of mastitis and days open in the investigated time intervals (Table 3). An interesting result is that in the same month of AI and 1 month before, we observed a higher number of somatic cells (*p* < 0.05) in the milk of the non-pregnant cows from the CH group than in the milk of the pregnant cows from the same group (Table 4).

**Table 3:** Timing of mastitis and its effect on pregnancy rate after the first artificial insemination (AI), number of services per calving (NSC), and days open.

Time of mastitis (days postpartum)	*n*	Conception rate at first AI (%)	NSC	Days open (d)
0–21	6	50^a,b^	2	100
22–42	9	44.4^b^	2.1	106
43–63	12	41.7^b^	1.6	108
64–84	18	38.8^b^	1.7	106
85–100	12	58.3^a^	1.3	117
Average mastitis cows until 100	58	44.8^b^	1.7	109
Total mastitis cows	146	41.7^b^	1.8	112
SEM		2.3	0.04	4.3
*p*-value		0.05	0.1	0.09

Different superscripts (^a,b^) in the same column indicate significant difference (*p* < 0.05).

**Table 4:** Somatic cells count of the pregnant and non-pregnant dairy cows from the CH group.

Reproductive status	SCC-0	SCC-1	SCC-2	SCC-3	SCC-4	SCC-BD	SCC-AC
Pregnant cows	235.6 ± 39.2^b^	323.5 ± 53.8^b^	194.6 ± 52.1	152.4 ± 41.1	160.7 ± 57.1	472.7 ± 74.6^a^	154.1 ± 39.5
Non-pregnant cows	470.3 ± 95^a^	465.5 ± 95.6^a^	231.0 ± 60	176 ± 36.6	145.4 ± 41.7	387.3 ± 83.7^b^	227.5 ± 72.8

SCC = SCC (multiplied by 10^3^ cells/mL).

SCC-0 = somatic cell count in the month of AI.

SCC-1 = somatic cell count before 1 month of the AI.

SCC-2 = somatic cell count before 2 month of the AI month.

SCC-3 = somatic cell count before 3 month of the AI month.

SCC-4 = somatic cell count before 4 month of the AI month.

SCC-BD = somatic cell count before dry.

SCC-AC = somatic cell after calving.

Different superscripts (a,b) in the same column indicate significant difference (*p* < 0.05).

According to the odds ratio analysis, the interaction between clinical mastitis and reproduction had a significant influence on the conception rate at first service. This means that cows from the CH group were 1.9 times more likely to remain pregnant compared to the CM group (Table 5).

**Table 5:** Odds ratios of the conception rate at first service variables included in the final logistic regression model (*n* = 473).

Factor	Class	*n*	% pregnancy	Odds ratio	95% confidence interval	*p*
Mastitis	CH group	190/327	58.1	Reference		
	CM group	61/146	41.7	1.9	1.3–2.9	<0.001

*R*^2^ Nagelkerke = 0.15.

The reproduction simulation program used in this experiment showed that the NPV of the CM group was US$-153.4/CM cow/year lower than that of the CH group. This study included the decrease in pregnancy rates at first service in the CM group compared to the CH group by approximately 16.5%, which can cause a loss of US$3,577/year just for the impact on replacement costs, reproduction costs, and calf value (Table 6). Added to this economic loss is the high cost of treating clinical mastitis, which amounts to $30/cow/year, and the loss of milk for 5 days (waiting time for antibiotics), which can amount to $98.9/cow/year (Table 6).

**Table 6:** Contribution to net present value (US$/cow/year).

Items (US$/cow/year)	CH group	CM group	Difference
Net present value	4,064.5	3,911.1	−153.4
Income over feed cost	4,306.2	4,313.9	7.7
Replacement cost	−280.6	−300.4	−19.8
Reproductive cost	−19.8	−25.4	−5.6
Calf value	58.7	51.9	−6.8
Mastitis treatment value	0	−30	−30
Milk loss value	0	−98.9	−98.9

## 4 Discussion

One of the most common diseases in dairy cows is clinical mastitis, which can affect reproductive parameters and increase costs by reducing reproduction. To the best of our knowledge, no study has investigated the financial impact of mastitis in dairy cows as an additional effect on reproduction. On the other hand, little is known about how clinical mastitis affects reproductive function in a high-yielding dairy farm in a temperate continental climate in Romania.

In our study, the conception rate at the first service, the conception rate at the third service, and the total conception rate were significantly affected in CM cows compared with CH cows but not the average days open which is significantly lower in CM cows. This means that although CM cows manage to conceive earlier, they do not have comparable reproductive performance to CH cows. The odds ratio analysis indicates that the CM group’s lower conception rate at first service is more likely than the CH cows. The reason for this could be insufficient follicular growth, anovulation caused by an impaired LH surge, or a decrease in estrogen synthesis leading to loss of estrus (16).

We observed a much lower conception rate at first service when cows expressed their clinical mastitis cases before AI in the interval of 22–84 days postpartum compared to the interval of 85–100 days postpartum and more. Conception rate at third insemination and total insemination conception rate were also impaired in CM cows compared with CH cows. The occurrence of clinical mastitis during early lactation and the days open period had detrimental effects on reproductive performance, possibly by altering the endocrine profile, follicular development (16), and probably uterine involution. To measure how GnRH, LH, cortisol, and progesterone (P4) are affected by inflammation, Battaglia et al. (39) administered intravenous endotoxin, a cell wall component of Gramme-negative bacteria that triggers an inflammatory response, to ewes. They also took simultaneous samples of jugular and pituitary portal blood at 10-min intervals and found lower GnRH pulse amplitude, lower concentrations of GnRH and LH, and increased concentrations of cortisol and P4. According to Darbon et al. (40), inflammation triggers the immune system and causes the release of cytokines that can block the effect of FSH on the formation of LH receptors in cultured rat granulosa cells as well as FSH-induced cAMP production. According to another study (41), cytokines released after endotoxin exposure inhibit GnRH by altering the production of nitric oxide, which blocks the pulsatile secretion of LH but not FSH. Therefore, alterations in LH and FSH activity or function may be one means by which mastitis affects reproductive function.

Systemic inflammation is another explanation, as it seems to play a role in balancing maladaptation and risk of disease or poor performance with adaptive/homeorhetic changes that support high milk yield. When inflammation is severe enough to cause systemic signs, fever and reduced feed intake also occur (42). The degree of trauma and bacterial contamination of the uterus or mammary gland are all related to the degree of systemic inflammation and lead to the release of proinflammatory cytokines (43). According to Horst et al. (44), inflammation induced by immune activation leads to reduced dry matter intake, which in turn causes hypocalcemia, increased levels of non-esterified fatty acids and ketosis. Their theory is that this contradicts the dogma of association, which claims that certain risk factors increase disease risk, reduce milk yield, or lower fertility. Instead, when present in excess, they represent the direct or indirect effects of inflammation (43). Furman et al. (45) proposed that the balance of signals representing pathogens [pathogen-associated molecular patterns (PAMP)] and tissue damage [damage-associated molecular patterns (DAMP)] modulate immune responses that can induce acute inflammation. However, DAMPs are thought to cause systemic inflammation associated with metabolic problems and more permanent tissue damage. This may be a good explanation for the low conception rate at the third service and total insemination conception rates for the CM group, considering that 60.2% of clinical mastitis cases occur at 100 days postpartum.

A very interesting aspect is that the non-pregnant cows from the CH group had more SCC in their milk around the time of artificial insemination and 1 month earlier than the pregnant cows. In the study by Rearte et al. (46), the conception risk in cows with high SCC before insemination was less affected than in the study by Lavon et al. (47), but the negative effect observed in severe cases of mastitis was almost the same. Based on the above data, we can hypothesize that the adverse effects of SCC on fertility appear to be more strongly associated with a reduction in the risk of conception than with the daily risk of service (46).

The study aimed to estimate as far as possible the additional costs associated with infertility as a side effect of clinical mastitis. The negative impact on the net present value consists of replacement costs, reproduction costs, calf value, mastitis treatment costs, and the value of milk loss. The most expensive losses are milk losses (US$14,439.4/farm/year) and those with mastitis treatment (US$4,380/farm/yr). The farm’s reproductive losses amount to approximately US$24/CM cow/year, resulting in a total net present value loss of US$3,577/farm/year. However, our estimate of the total net loss due to clinical mastitis on the farm studied is approximately US$22,396.4/farm/year.

The impact of mastitis on herds and the economy has been demonstrated in numerous studies (48–51). According to Huijps et al. (49) and Bonestroo et al. (12), the main factors influencing the economic impact of mastitis are the decrease in milk production due to clinical and subclinical cases, the disposal of milk, the cost of drugs to treat clinical cases, the labor costs associated with the treatment of clinical cases, the decrease in the milk selling price and the culling of animals. The decline in milk production and culling have the greatest financial impact on the total cost of mastitis according to Huijps et al. (49) and Bonestroo et al. (12). Nevertheless, the producer underestimates the decline in milk yield (49). In our study, the decline in milk yield of the farm is the primary factor influencing the NPV compared to the decline in reproductive function and the treatment costs of mastitis. However, the latter two parameters cannot be neglected as together they cause a net present value loss of approximately US$8,000/farm/year. Considering that the non-pregnant cows from the CH group had a high number of SCC compared to the pregnant cows, we can speculate that also in this case, there are additional costs to the reproductive losses, costs that we can hardly estimate, but we are sure that they add up to the losses leading to the NPV.

## 5 Conclusion

The effect of mastitis on reproduction appears to be associated with a lower chance of conception rather than the daily risk of services. Economically, the most expensive costs are those of mastitis treatment and milk loss; subsequent reproductive losses are less expensive than the initial ones. Our study highlights the importance of udder health in dairy farming. Improving udder health can lead to better reproductive performance and higher NPV for dairy farms.

## Data availability statement

The raw data supporting the conclusions of this article will be made available by the authors, without undue reservation.

## Ethics statement

All dairy cows were handled in accordance with the directives of the European Union on the protection of animals used for scientific purposes (Dir 2010/63/EU). The experimental protocol was approved by the Ethics Committee of the Faculty of Veterinary Medicine, University of Life Sciences, 700489 Iasi, Romania. Efforts were made to minimize animal handling and stress. The study was conducted in accordance with the local legislation and institutional requirements.

## Author contributions

AB: Conceptualization, Methodology, Supervision, Validation, Visualization, Writing – original draft, Writing – review & editing. S-IB: Conceptualization, Data curation, Investigation, Methodology, Resources, Software, Writing – original draft, Writing – review & editing. V-CF: Formal analysis, Supervision, Validation, Visualization, Writing – original draft, Writing – review & editing.
